# Perceived Stress and Sociodemographic Factors Among Saudi Women with Breast Cancer: A Cross-Sectional Study

**DOI:** 10.3390/jcm15031168

**Published:** 2026-02-02

**Authors:** Sahar Abdulkarim Al-Ghareeb, Ahmad Aboshaiqah, Mousa Yahia Asiri, Homoud Ibrahim Alanazi, Ahmad M. Rayani

**Affiliations:** 1 College of Nursing, King Saud University, Riyadh 11421, Saudi Arabia; 445205106@student.ksu.edu.sa (S.A.A.-G.); aaboshaiqah@ksu.edu.sa (A.A.); mosa-909@hotmail.com (M.Y.A.); 2 Nursing Department, King Saud University Medical City, Riyadh 12746, Saudi Arabia; hoalanazi@ksu.edu.sa; 3 Community and Psychiatric Mental Health Nursing Department, College of Nursing, King Saud University, Riyadh 12372, Saudi Arabia

**Keywords:** breast cancer, perceived stress, sociodemographic factors, Saudi Arabia

## Abstract

**Background:** and objective: Globally, breast cancer (BC) raises global health concerns, being the most common cancer. Women with BC experience a significant increase in perception of stress. Therefore, this study aims to evaluate the stress levels and associated sociodemographic and clinical factors among BC women in Saudi Arabia. **Methods:** A cross-sectional study was conducted between January and May 2025. Women diagnosed with BC, who were at least 18 years old, were recruited conveniently from outpatient and inpatient departments in King Fahad Specialist Hospital, Dammam, Saudi Arabia. Data were collected in the Arabic language through self-reported questionnaires, including sociodemographic/clinical characteristics and the Cohen’s Perceived Stress Scale. The data were analyzed using the Statistical Package for the Social Sciences (SPSS) version 27. **Results:** A total of 200 participants were included in the study. The mean stress perception score was 26.52 ± 7.34. A high proportion (71.5%) of the sample reported elevated stress. A significant association was observed between age and stress levels. Most women aged 20–40 and 41–60 reported high stress, compared to women in the 61–80 age group (*p* = 0.003). Among all predictors, age was the only variable significantly associated with stress scores. Increasing age was associated with lower stress levels (B = −0.179, *p* = 0.013), indicating that younger participants tended to report higher stress. This corresponds to an adjusted decrease of approximately 1.8 points in the PSS-10 score per 10-year increase in age. Although participants with Stage IV cancer showed higher stress scores compared to those with Stage I cancer, this association approached but did not reach statistical significance (*p* = 0.054). **Conclusions:** This study highlights the substantial psychological burden experienced by women living with BC in Saudi Arabia. The majority of participants reported high levels of perceived stress. Younger women were particularly vulnerable to elevated stress. These findings highlight the need for targeted psychosocial support within oncology care to improve emotional well-being and quality of life.

## 1. Introduction

Globally, cancer incidence continues to rise, with breast, lung, and prostate cancers being the most commonly diagnosed types [1,2]. More recently, literature shows that approximately 2.3 million women across 152 countries were newly diagnosed with breast cancer (BC) in 2022, making it the most common cancer in women globally, while BC accounted for a substantial portion of the 670,000 cancer-related deaths that year [1,3]. In the Kingdom of Saudi Arabia, the burden of BC is also rising [4,5]. A national time-series analysis reported that age-standardized incidence rates increased from 15.4 per 100,000 women in 1990 to 46.0 per 100,000 in 2021, underscoring a substantial escalation over three decades [1,4]

In Saudi Arabia, BC remains a major public health concern, contributing to a substantial number of deaths each year. Women diagnosed with BC often undergo a range of therapeutic treatments, which can have significant physical, emotional, and psychological challenges [6,7,8]. Evidence from international research shows that many women experience considerable stress following their diagnosis [9]. Perceived stress is a psychological issue, which is defined as the subjective individual’s interpretation and sensation of life events that are beyond their abilities and coping, regardless of the actual situation [10]. The psychological dysfunction is associated with many factors, including social, economic, and work-related factors, also decreases adherence to the physician’s instructions, diminished wound healing leading to prolonged hospitalizations, and recurrent hospital visits, which all alter their quality of life [11,12]. Therefore, it is crucial to investigate factors influencing perceived stress among women with BC to take measures to diminish their psychological problems.

International studies have examined the influence of sociodemographic factors on perceived stress among women with BC, with several reporting an inverse association between age and stress, indicating that older women experience lower stress levels [13,14]. However, other evidence suggests that older age may be associated with greater psychological distress [15], highlighting inconsistencies in the literature. Marital status has also been identified as a relevant factor, with divorced or widowed women reporting higher stress levels compared to other groups [16]. In Arabic countries, research has similarly documented substantial psychological distress among women with BC. Previous studies have explored psychological well-being and quality of life, including evaluations of support programs qualitative assessments of distress experiences [7,17,18], investigations of psychological resilience [17] and systematic reviews focusing on quality of life among Saudi women with BC [18].

Although stress perception is influenced by cultural perspectives and religious factors, Saudi Arabia has distinct cultural expectations and stigma around cancer that may elaborate the perception of stress differently than the Western context [19]. Understanding women’s personal feelings of stress is essential. Although several Saudi studies have assessed the psychological stress and quality of life among Saudi women with BC [17,18,19], there is a lack of quantitative studies that have solely examined perceived stress among Saudi women with BC. Moreover, the sociodemographic impact on perceived stress remains unexplored, specifically addressing the mediating role of women’s employment status. This study is the one of the few quantitative studies to quantify perceived stress utilizing a validated perceived stress scale among Saudi BC women [20]. Therefore, this study aims to evaluate the level of perceived stress and its relationship with sociodemographic factors among women with BC in Saudi Arabia. While several studies have documented psychological stress among women with BC in Saudi Arabia and neighboring regions [6,7,8,9], most have focused on prevalence estimates of anxiety or depression, often using heterogeneous measurement tools. Less attention has been paid to perceived stress as a continuous psychological construct and to how stress varies across sociodemographic subgroups. The present study addresses this gap by employing the validated Arabic version of the Perceived Stress Scale (PSS-10) to examine stress distributions and gradients across age, education, marital status, and employment among Saudi women with BC. By moving beyond prevalence alone and exploring subgroup differences in stress levels, this study provides a more nuanced understanding of psychosocial vulnerability within this population

By highlighting, the influence of sociodemographic and clinical characteristics, the present findings help reconcile these seemingly contradictory results. These insights can inform oncology organizations in designing more targeted supportive care strategies, including counseling and stress-management programs tailored to specific patient profiles. Furthermore, the results underscore the value of integrating routine psychological screening within oncology settings to identify women at higher risk of elevated stress, thereby supporting evidence-based psychosocial care planning at both national and international levels. Therefore, this study aimed to assess the stress levels, associated sociodemographic, and clinical factors among BC women in Saudi Arabia

## 2. Methods

A cross-sectional quantitative study was conducted in King Fahad Specialist Hospital, Dammam (KFSH-D), Eastern Province, Saudi Arabia. KFSH-D is an oncology hospital treating different oncology cases with a high flow of BC patients. Eligible participants were recruited through convenience sampling from the outpatient (OPD) and inpatients departments. To be eligible for the study, we included women who BC as a primary diagnosis, aged 20 to 80 years, women with ongoing BC treatment, and accepted to participate. Women with a documented diagnosis of a pre-existing psychiatric or psychological disorders and do-not-resuscitate (DNR) status were excluded to avoid confounding effects on perceived stress and to ensure that responses primarily reflected cancer-related experiences. Information on psychiatric diagnoses and DNR status was ascertained through review of medical records at the time of recruitment. Participants with a Do Not Resuscitate (DNR) order were also excluded from the study.

### 2.1. Ethical Consideration

All study procedures were performed in compliance with the Declaration of Helsinki guidelines for human research, Institutional Review Board (IRB) approval was obtained from the KFSH under the number EXT0449. Prior to data collection, written informed consent was secured from participants who expressed interest in participating. In addition, the participants were provided clear instructions about the study purpose, benefits, potential risks, components of the scale, method for completing the scale, and duration of the study, which will be completed at one time. Furthermore, the participants were assured that their confidentiality would be maintained, no identification data would be collected, their participation was voluntary, and they had the right to withdraw at any time, which would not affect the given care. The data was stored privately and will be retained for two years.

### 2.2. Sample Size

**Figure 1:**
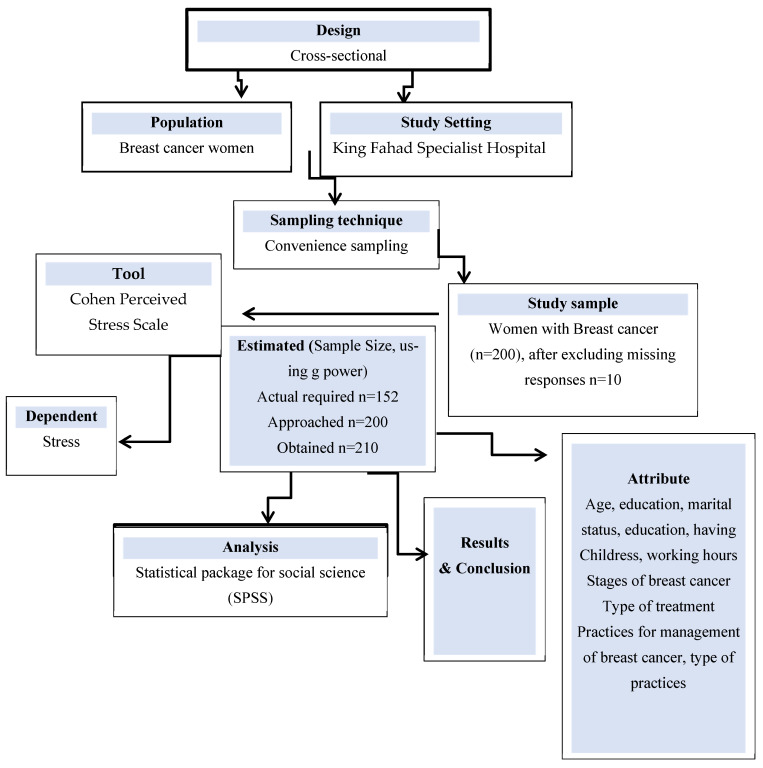
Overview of research design.

The required sample size for this study was determined using an a priori power analysis conducted with G*Power (version 3.1). Assuming a medium effect size (r = 0.30), a two-tailed test, a significance level of α = 0.05, and a desired statistical power of 0.80, the analysis indicated that a minimum of 138 participants was required. After inflating the estimate by 10% to account for potential non-response or missing data, the minimum target sample size was 152 participants. Furthermore recruitment continued until we reached around 200 participants to improve statistical precision, enable multivariable analyses, and ensure adequate representation across sociodemographic subgroups. However, a total of 2010 responses were received during the study period (Figure 1).

### 2.3. Study Tool

The study questionnaires include two sections, where section one-collected socio-demographic and clinical characteristics, and section two deals with the Cohen Perceived Stress Scale (PSS). The Socio-demographic and Clinical Characteristics are further divided into two sections: the sociodemographic section, which contains participants’ age, marital status, whether they have children, level of education (low, moderate, and high), and employment status (full time; working least 20 h per week, part time; working less than 20 h per week, un-employee). The second section is the clinical characteristics: time of cancer diagnosis, stage of cancer, type of surgical procedure, and whether they attended any relaxation or meditation strategies. Section two of the study deals with assessing the PSS, which is adopted from earlier studies to examine the extent to which individuals perceive life situations as stressful and uncontrollable over the past month. The scale includes 10 items rated on a five-point Likert response format ranging from 0 (never) to 4 (very often). Positively phrased items are reverse-scored (0 becomes 4, 1 becomes 3, and so on). The total score was computed by summing all the items. The higher the score given, the higher the level of perceived stress [4,20].

Perceived stress was measured using the PSS-10. Scores were categorized into low (0–13), moderate (14–26), and high (27–40) stress based on the original scale guidelines [4,20]. These cut-offs were chosen to facilitate interpretation and comparison with previous studies in similar populations [4]. However, for inferential analyses, categories were collapsed into high versus low/moderate stress to ensure adequate cell sizes and stable statistical estimates.

After the initial draft of the questionnaire, it was subjected to expert review for content, flow, and understanding of the questionnaire. The expert opinion revealed no changes. Later, a pilot study was carried out among 30 randomly selected women with BC. The reliability of the PSS was determined by assessing the Cronbach alpha, which was 0.83, suggesting that the good internal consistency reliability for the study. However, the original Arabic scale shows adequate concurrent and convergent validity. Moreover, the subscales of the PSS revealed good internal consistency [20].

### 2.4. Data Collection Procedure

Prior to obtaining the ethical approval from the institutional review boards (IRB), a research team was formulated and consisted of the primary researcher and an oncology nurses to explain the research purpose, eligible participants, components of the questionnaire, method of data collection, importance of securing the privacy of the study participants, data entry, and storage. The participants were recruited during their follow-up on OPD and their admissions. They were assured of their privacy and anonymity. Those who expressed interest and consented to participate were requested to complete the paper-based self-reported questionnaire at one time lasted for 15–20 min. Data was entered and coded in an Excel sheet before being exported to SPSS. The mean overall score for the perceived stress was computed by combining all 10 items and was divided into low stress, where participants who scored less than 20% of the total mean score, and scores of 21 and above were considered high stress among respondents.

### 2.5. Data Analysis

Data were analyzed using statistical package for social science (SPSS) version 27. Descriptive statistics were used to summarize participant characteristics and study variables. Categorical variables were reported as frequencies and percentages, while continuous variables were summarized using means and standard deviations. The primary outcome, perceived stress, was analyzed in two forms. The total PSS-10 score was treated as a continuous variable for regression analyses, while stress severity categories (low/moderate vs. high) were used for descriptive purposes and group comparisons (bivariate analyses). For bivariate analyses addressing group differences in stress categories, chi-square tests were applied. These analyses addressed research questions related to differences in stress prevalence across sociodemographic and clinical subgroups. For multivariable analyses addressing predictors of stress magnitude, linear regression modelling was conducted using the continuous PSS-10 score as the dependent variable. Predictor variables were coded as follows: age and time of diagnosis were treated as continuous variables; education level, marital, employment status, working hours, cancer stage, type of therapy, and type of surgery were entered as multi-level categorical variables using indicator (dummy) coding; binary variables (e.g., having children, surgery status, presence of other cancer, engagement in stress-related practices) were coded as yes/no. The distribution of the continuous PSS-10 score was assessed for normality using the Shapiro–Wilk test, which did not indicate a significant deviation from normality (*p* = 0.350). Based on this assessment, linear regression analysis was considered appropriate. A multivariable linear regression model was constructed to examine factors independently associated with perceived stress scores while adjusting for potential confounders. Regression results are presented as unstandardized beta coefficients with corresponding 95% confidence intervals and *p*-values. Statistical significance was set at *p* < 0.05.

The primary outcome of the study was the continuous PSS-10 total score, and the primary analysis was the multivariable linear regression model examining adjusted associations between perceived stress and demographic and clinical predictors. Bivariate analyses (e.g., chi-square tests and unadjusted comparisons) were conducted as exploratory analyses to describe patterns in the data and to inform subsequent multivariable modelling. Given their exploratory purpose, no formal adjustment for multiple comparisons was applied, and findings from these analyses were interpreted cautiously.

## 3. Results

During the study period, 210 responses were received. After excluding incomplete questionnaires (*n* = 10), data from 200 Saudi women diagnosed with BC were included in the analysis, resulting in a response rate of 95.2%. The mean age of participants was 47.7 ± 9.24 years. Most women were between 41 and 60 years of age (66%), followed by those aged 20–40 years (24%), while 10% were aged 61–80 years. Regarding educational attainment, 34% of participants held a bachelor’s degree, and 28% had completed high school. The majority of women were married (72.5%) and had children (77%). Employment levels were relatively low, with only 28% of participants reporting paid employment; among these, 21% worked full-time and 7% worked part-time. A detailed summary of the participants’ demographic characteristics is provided in Table 1.

In this study, the mean time since BC diagnosis was 21.3 ± 39.9 months (median: 10 months). More than half of the women had been diagnosed within the preceding 6–12 months, while 19.5% were diagnosed more recently. Concerning stages of BC, 21.5% of the women were diagnosed with stage 2 BC. Regarding treatment, 44.5% of the women underwent more than one therapy, while 38% of them were treated by chemotherapy during the study period. Overall, in this study, 69% of women underwent surgery since diagnosis, with nearly half of those surgeries completed within the past six months (47.5%) (Table 2). Among surgical procedures, complete mastectomy (34%) was the most common, nipple-sparing procedures (6%), and breast reconstruction (3%) were less frequent. Only 5% of participants reported having other types of cancer in addition to BC. More than half (59.2%) engaged in some form of supportive or wellness practice. The most frequently reported activities included self-care and mindfulness (35%), followed by multiple combined practices (38.5%), as shown in Table 2.

Saudi women in the current study reported a wide range of perceived stress-related experiences over the past month. When asked how often unexpected events upset them, about one-third (31.5%) said this happened sometimes, while 21% experienced it a lot and 10.4% reported it always. Feelings of nervousness or stress were also common among BC women. Additionally, 16.5% felt stressed most of the time. In this study, many participants reported low confidence in managing personal problems, with 32% choosing never and 30.1% selecting rarely confident. When asked whether things were going their way, 40.5% reported this occurred sometimes, but only 3.5% felt it happened most of the time. Difficulty coping with responsibilities appeared common, as 29.5% struggled sometimes, and 17% struggled a lot. Finally, when asked whether difficulties felt overwhelming, 22% experienced this sometimes, 21.5% a lot, and 24% reported they never felt unable to overcome their difficulties. Table 3. Shows frequency of responses towards Perceived Stress.

**Figure 2:**
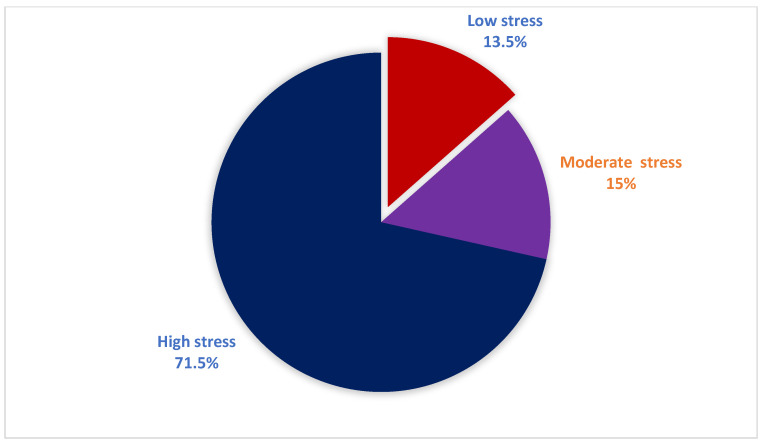
Levels of stress among women with breast cancer.

In this study, 71.5% of women reported high levels of perceived stress, as shown in Figure 2.

A significant association was observed between age and stress levels. Most women aged 20–40 and 41–60 reported high stress, compared to women in the 61–80 age group (*p* = 0.003). Other demographic factors, including education level, marital status, having children, employment status, and weekly work hours, did not demonstrate a significant association with stress levels, as shown in Table 4.

Table 5 explores whether different clinical characteristics of the participants are linked to their stress levels. Overall, most clinical factors did not show a statistically significant relationship with stress. For example stress levels did not differ across the stages of BC (*p* = 0.070), type of therapy received (*p* = 0.946), undergoing surgery (*p* = 0.572), timing (*p* = 0.636), type of breast surgery (*p* = 0.803), having another type of cancer (*p*= 0.100), practicing stress-management or wellness activities (*p* = 0.283). However, when examining the specific types of practices, a significant association emerged (*p* = 0.037). Participants who engaged in self-care and mindfulness accounted for 35.7% of the high-stress group, while physical activity was more common among highly stressed individuals (13.3%) compared with those with low stress (3.5%). Many participants used more than one stress-management approach, but high stress remained common even in this group (37.8%) Table 5.

Additionally, multiple linear regression analysis was conducted to assess the association between mean stress scores and selected demographic and clinical characteristics of the participants (Table 6). The overall regression model explained 16.3% of the variance in stress scores (R^2^ = 0.163); however, the adjusted R^2^ was very low (0.008), indicating that the predictors collectively contributed little to explaining stress levels. The ANOVA results showed that the model was not statistically significant (F = 1.053, *p* = 0.401), suggesting that, overall, the independent variables did not significantly predict mean stress scores.

Among all predictors, age was the only variable significantly associated with stress scores. Increasing age was associated with lower stress levels (B = −0.179, *p* = 0.013), indicating that younger participants tended to report higher stress. This corresponds to an adjusted decrease of approximately 1.8 points in the PSS-10 score per 10-year increase in age.

Educational level, marital status, having children, working hours per week, time since diagnosis, cancer stage, type of therapy, type of surgery, and participation in stress-management practices were not significantly associated with stress scores when compared to reference category (all *p* > 0.05). Although participants with Stage IV cancer showed higher stress scores compared to those with Stage I cancer, this association approached but did not reach statistical significance (*p* = 0.054).

Overall, these findings indicate that while age plays a meaningful role in influencing stress levels among participants, the remaining demographic and clinical factors examined in this model did not independently contribute to variations in perceived stress (Table 6).

## 4. Discussion

The psychological well-being of women diagnosed with BC plays a crucial role in shaping their overall health outcomes. Although the disease itself is a major source of emotional strain, additional stress arising from social, economic, and demographic challenges can further intensify their stress and negatively influence recovery. The present study offers valuable insight by highlighting how these factors contribute to perceived stress, thereby supporting efforts to enhance stress-management strategies for this group. The current findings revealed that 71.5% of women with BC were found to have high stress levels. Additionally, 31.5% of these women reported feeling upset always or a lot because of unexpected events, while 23% felt unable to control important aspects of their lives. Furthermore, 44% of them felt nervous or stressed. These findings highlight a significant psychological burden in our sample, which appears to be greater than in many previous international reports and consistent with the higher end of stress prevalence in regional studies. These findings were consistent with earlier findings published in other countries [6,21,22,23]. For instance, Alagizy et al. in 2020 assessed prevalence and associated psychosocial factors of mental disorders, and perceived stress among BC patients among Egyptian women with BC and revealed 78.1% of women suffering from stress [6]. Similarly, another study among BC women in Poland reported 54.9% suffering from stress, particularly feelings of fear and anxiety, accompanied 30.5% of the women, and 24.7% [21]. In another study, a systematic review and meta-analysis of 34 studies aimed to assess the prevalence and correlates of psychological stress in patients with BC revealed psychological stress in 50% [22]. In Iran, the prevalence of stress among BC Iranian women was 51.9% [23]. The differences in the prevalence of stress in the current literature and earlier are likely attributable to sampling (hospital vs. population), timing (near diagnosis/active treatment), the instrument used, and cultural health-related factors. These results underscore the substantial psychosocial burden among Saudi women with BC and the need for routine screening and culturally sensitive psychosocial interventions.

Although BC presents challenges that extend beyond medical treatment, many survivors continue to experience emotional issues such as stress and worry, which can significantly affect daily functioning and overall well-being [23]. Complex treatments for BC, including surgery, chemotherapy, radiation, and hormone therapy, may contribute to psychological stress [23]. These emotional and cognitive challenges can make routine activities more difficult, increasing perceived stress levels and highlighting the need for targeted psychosocial support for women undergoing cancer treatment [23,24,25].

In the present study, women’s age and coping practices were associated with variations in reported stress levels in the unadjusted analyses. Higher levels of stress were more commonly reported among women aged 20–40 and 41–60 years compared with those in the older age group. This pattern may reflect differences in life circumstances, as older women may have fewer responsibilities related to employment or household demands and may spend more time at home. In contrast, younger and middle-aged women are often managing multiple roles, which may contribute to greater perceived stress. Differences were also observed in coping practices across stress categories. Women who reported engaging in self-care and mindfulness activities comprised a substantial proportion of the high-stress group, while physical activity was reported more frequently among women with high stress than among those with lower stress levels. Many participants indicated using more than one strategy to manage stress, suggesting that coping behaviors often overlap rather than occur in isolation. When these variables were examined in the adjusted multivariable model, however, age remained the only factor independently associated with perceived stress. The associations observed for coping practices did not persist after adjustment, indicating that these findings should be interpreted as descriptive and exploratory. Overall, the most consistent and robust finding of this study was the inverse relationship between age and perceived stress, which remained significant after controlling for relevant sociodemographic and behavioral factors. This highlights age as an important consideration in understanding stress perceptions, particularly among younger individuals.

On the other hand, women with a bachelor’s degree constituted the largest share of the high-stress group, followed by those with a high school education. Participants with lower educational levels were less represented in the high-stress category. Although no statistically significant associations were found between stress levels and education level in the current study. While earlier studies reported mixed findings [16,26,27]. For example a recent study by Eker et al., in 2024 reported that those with higher level of education revealed higher stress, which is similar to current findings [27]. Similarly, another study among cancer patients found that higher education was associated with more frequent reports of stress [26]. Then again Vafaei et al., in 2023 among BC women’s revealed that lower educational level express elevated perceived stress which is oppose to the current findings [16]. This could be because educated patients are more conscious of the burden and consequences of the illness. Women with greater education are frequently more informed about cancer, treatment options, possible side effects, and long-term prognosis. This improved understanding can lead to heightened anxiety about prognosis, recurrence, and treatment problems.

Other demographic factors, including marital status, having children, employment status, and weekly work hours, did not demonstrate a significant association with stress levels. Similar findings were reported in earlier studies. For example, Hessami et al. in 2025 found no significant difference in stress levels by marital status [23]. Employment status plays a significant role in predicting the incidence of perceived stress among women with BC. An analytical study revealed that the effects of employment status depend on individuals’ economic status [28]. While another study revealed that the unemployed women with BC reported a higher level of perceived stress [27] compared to working individuals [27]. Employed BC patients may perceive stress differently due to challenges to balance treatment and job requirements (Kong et al., 2018) [29], while those unemployed may face more economic and social barriers that increase stress (Yang et al., 2024) [30]. Thus, employment status partly explains (mediates) the relationship between sociodemographic and stress in this group. Although in the current study, the number of employed women was very small, and they often received strong emotional and practical support from family members, which can buffer stress, which might be the reason for the differences.

Furthermore, studies show that social support is a stronger predictor of stress than employment among Middle Eastern BC patients [31,32,33]. These differences may stem from cultural, methodological, or contextual differences, highlighting the need for more standardized research. In this study, most clinical variables, including cancer stage, type of therapy, undergoing surgery, timing of therapy, type of breast surgery, and comorbid cancer history, did not show statistically significant associations with perceived stress (*p* > 0.05). Several explanations may account for these null findings. First, the absence of significant associations could reflect a true lack of effect, suggesting that stress levels may be influenced more strongly by psychosocial factors rather than the clinical characteristics measured. Second, the limited sample size in some subgroups may have reduced statistical power, making it more difficult to detect small but potentially meaningful differences. Third, dichotomizing the PSS into high and low categories, while clinically interpretable, may have diminished variability and obscured subtle associations. Despite these null associations, examining specific stress-management practices revealed significant patterns: participants engaging in self-care and mindfulness were more represented among the high-stress group (35.7%), while physical activity was more common among highly stressed participants (13.3% vs. 3.5% in low stress). These findings suggest that stress coping behaviors are relevant to perceived stress and may vary in effectiveness. Overall, the results underscore the complexity of stress experiences in BC patients and indicate that both clinical and behavioral factors warrant further exploration.

The present study contributes to the existing literature by extending prior Saudi and regional research in several important ways. Unlike earlier studies that primarily reported prevalence rates of anxiety or depression [13,15,16], this study assessed perceived stress using the validated Arabic PSS-10, allowing for culturally appropriate measurement and comparison of stress score distributions across sociodemographic groups. The observed stress levels in our cohort were comparable to those reported in regional studies of BC patients; however, the distribution of stress across age groups revealed meaningful gradients that are often obscured when psychological outcomes are reported solely as categorical prevalence figures. For example, while previous Saudi studies have documented high overall psychological stress among women with BC [5,6,7], few have quantified differences in stress magnitude between sociodemographic subgroups. Our findings suggest that perceived stress is not uniformly distributed, supporting international evidence that social and economic contexts shape stress experiences in cancer survivorship. By highlighting effect sizes and subgroup patterns rather than only prevalence, this study adds depth to the understanding of psychosocial burden among Saudi women with BC and provides evidence that may inform targeted psychosocial interventions. Although the absence of significant associations for other variables in the adjusted model may reflect the cross-sectional design, shared variance among predictors, or unmeasured contextual factors, and does not preclude their potential relevance in other settings or study designs.

This study has some limitations, which must be acknowledged. First, the bivariate analyses involved multiple comparisons and were exploratory in nature; therefore, statistically significant findings from these analyses may reflect chance associations and should be interpreted with caution. The primary inferences of the study are based on the adjusted multivariable model. Secondly, the sample included in this study is limited to a single hospital and from a single region in Saudi Arabia. Therefore, these findings cannot be generalized to the entire community of BC women in the Saudi population. Future studies could aim to include a larger and more diverse sample, including those from multiple hospitals across different regions in the country, to enhance representativeness even further. Additionally, all data were collected through convenience sampling, which may also result in bias and is considered a limitation. Another possible limitation of this study is the binary (yes/no) assessment of coping practices and support resources, which restricted our ability to examine these factors in greater detail. The type, frequency, and intensity of coping behaviors or support mechanisms were not captured, limiting further stratification. In addition, another possible limitation is that multivariable regression analysis was employed, formal diagnostic assessments of multicollinearity and linearity assumptions were not performed, which should be considered when interpreting the adjusted estimates. Future studies should utilize validated multidimensional coping and social support instruments to enable more nuanced analyses. However, this study provided a unique opportunity to inform healthcare authorities on implementing adequate measures to deliver healthcare services in the holy cities of Saudi Arabia.

## 5. Conclusions

This study reveals a significant burden of perceived stress among women with BC, with over two-thirds of participants reporting elevated stress levels. Age was the only factor found to be significantly associated with perceived stress, showing a consistent inverse relationship in both correlation and regression analyses. Younger and middle-aged women experienced higher stress levels compared to older participants, suggesting that stress perception decreases with age. In contrast, working hours and all examined clinical characteristics were not significantly associated with stress, indicating that perceived stress in this population may be more strongly linked to sociodemographic factors rather than clinical variables. These findings emphasize the importance of incorporating age-sensitive psychological assessment and psychosocial support into routine oncology care. While the cross-sectional design limits causal interpretation, the results underscore the need for regular stress screening, especially among younger women.

## Figures and Tables

**Table 1:** Sociodemographic characteristics among Saudi Women with Breast Cancer (*n* = 200).

Variables	Frequency (*n*)	Percentage (%)
Age		
20–40	48	24%
41–60	132	66%
61–80	20	10.0%
**Education level**		
Not educated	8	4.0%
Primary	25	12.5%
Secondary	17	8.5%
High school	56	28%
Diploma	23	11.5%
Bachelor	68	34%
Master	3	1.5%
**Marital status**		
Single	29	14.5%
Married	145	72.5%
Widow	11	5.5%
Divorced	15	7.5%
**Having children**		
Yes	154	77%
No	46	23%
**Are you employed**		
Yes	56	28%
No	144	72%
**Employment hours (per week)**		
Full duty (more than 20 h)	42	21%
Part-time (less than 20 h)	14	7.0%
Not employed	144	72%

**Table 2:** Clinical characteristics of the women with breast cancer (*n* = 200).

Variables	Frequency (*n*)	Percentage (%)
**Time of Diagnosis (months)**		
1–5	39	19.5
6–12	115	57.5
13–24	16	8.0
>25	30	15
**Stages of Breast cancer**		
Stage-1	41	20.5
Stage-2	43	21.5
Stage-3	39	19.5
Stage-4	15	7.5
I don’t know	62	31
**Type of therapy taken**		
Chemotherapy	76	38
Radiotherapy	13	6.5
Hormonal	22	11
More than one therapy	89	44.5
**Type of surgery**		
Complete mastectomy	69	34.5
Partial mastectomy	30	15
Nipple sparing	12	6
Breast re construction	5	2.5
Not done breast cancer surgery	64	32
More than one surgery	20	10
**Undergone surgery**		
No	62	31%
Yes	138	69%
**Time of surgery**		
<6 months	95	47.5%
>6 months	45	22.5%
No surgery	60	30%
**Other types of cancer**		
No	190	95.0%
Yes	10	5.0%
**Did you have practices**		
No	82	41%
Yes	118	59%
**Type of Practices**		
Physical exercise and activity	21	10.5%
Social support	21	10.5%
Self-care and mindfulness	70	35%
More than one	77	38.5%
Other	11	5.5%

**Table 3:** Participant’s frequency of responses towards Perceived Stress (*n* = 200).

Variables	Frequency (*n*)	Percentage (%)
**In the past month, how often have you been upset because of something that happened unexpectedly?**		
Never	43	21.5%
Rare	31	15.5%
Sometimes	63	31.5%
A lot	42	21%
Always	21	10.5%
**In the past month, how often have you felt unable to control the important things in your life?**		
Never	49	24.5%
Rare	52	26%
Sometimes	53	26.5%
A lot	35	17.5%
Always	11	5.5%
**In the past month, how often have you felt nervous or stressed?**		
Never	21	10.5%
Rare	28	14%
Sometimes	63	31.5%
A lot	55	27.5%
Always/most of the time	33	16.5%
**In the past month, how often have you felt confident about your ability to handle personal problems?**		
Never	64	32%
Rare	62	31%
Sometimes	53	26.5%
A lot	15	7.5%
Always	6	3.0%
**In the past month, how often have you felt that things were going your way?**		
Never	38	19%
Rare	43	21.5%
Sometimes	81	40.5%
A lot	31	15.5%
Always	7	3.5%
**In the past month, how often have you found that you could not cope with all the things you had to do?**		
Never	36	18%
Rare	66	33%
Sometimes	59	29.5%
A lot	34	17%
Always/most of the time	5	2.5%
**In the past month, how often have you been able to control irritations in your life?**		
Never	38	19%
Rare	62	31%
Sometimes	68	34%
A lot	22	11%
Always/most of the time	10	5.0%
**In the past month, how often have you felt that you were on top of things?**		
Never	50	25%
Rare	53	26.5%
Sometimes	66	33%
A lot	19	9.5%
Always/most of the time	12	6.0%
**In the past month, how often have you been angry because of things that happened that were outside of your control?**		
Never	27	13.5%
Rare	50	25%
Sometimes	55	27.5%
A lot	48	24%
Always/most of the time	20	10.0%
**In the past month, how often have you felt that difficulties were piling up so high that you could not overcome them?**		
Never	48	24%
Rare	48	24%
Sometimes	44	22%
A lot	43	21.5%
Always/most of the time	17	8.5%

**Table 4:** Association between the stress levels and participants’ demographics (*n* = 200).

Variables	Stress Levels		*p* Value
	Low/Moderate *n* (%) 28.5%	High *n* (%) 71.5%	
**Age**			0.003
20–40	10 (17.5)	38 (26.6)	
41–60	35 (61.4)	97 (67.8)	
61–80	12 (21.1)	8 (5.6)	
**Education**			0.061 *
Not educated	5 (8.8)	3 (2.1)	
Primary	7 (12.3)	18 (12.6)	
Secondary	9 (15.8)	8 (5.6)	
High school	11 (19.3)	45 (31.5)	
Diploma	6 (10.5)	17 (11.9)	
Bachelor	18 (31.6)	50 (35.0)	
Master	1 (1.8)	2 (1.4)	
**Marital status**			0.198 *
Single	4 (7.0)	25 (17.5)	
Married	47 (82.5)	98 (68.5)	
Widow	3 (5.3)	8 (5.6)	
Divorced	3 (5.3)	12 (8.4)	
**Having children**			0.271
Yes	47 (82.5)	107 (74.8)	
No	10 (17.5)	36 (25.2)	
**Are you employed**			0.383
Yes	13 (22.8)	43 (30.1)	
No	44 (77.2)	100 (69.9)	
**Employment hours (per week)**			0.591
Full duty (more than 20 h)	9 (15.8)	33 (23.1)	
Part-time (less than 20 h)	4 (7.0)	10 (7.0)	
Not employed	44 (77.2)	100 (69.9)	

* Fisher exact test.

**Table 5:** Association between the stress levels and participants’ Clinical characters (*n* = 200).

Variables	Stress Levels		*p* Value
	Low *n* (%)	High *n* (%)	
**Stages of Breast cancer**			0.070 *
Stage-1	13 (22.8)	28 (19.6)	
Stage-2	13 (22.8)	30 (21)	
Stage-3	10 (17.5)	29 (20.3)	
Stage-4	0 (0.0)	15 (10.5)	
I don’t know	21 (36.8)	41 (28.7)	
**Type of Therapy**			0.946 *
Chemotherapy	22 (38.6)	54 (37.8)	
Radiotherapy	4 (7.0)	9 (6.3)	
Hormonal	7 (12.3)	15 (10.5)	
More than one therapy	24 (42.1)	65 (45.5)	
**Undergone surgery**			0.572
Yes	16 (28.1)	46 (32.2)	
No	41 (71.9)	97 (67.8)	
**Time of surgery**			0.636
<6 months	30 (52.6)	65 (45.5)	
>6 months	11 (19.3)	34 (23.8)	
No surgery	16 (28.1)	44 (30.8)	
**Type of surgery**			0.803 *
Complete mastectomy	20 (35.1)	49 (34.3)	
Partial mastectomy	7 (12.3)	23 (16.1)	
Nipple sparing surgery	3 (5.3)	9 (6.3)	
Breast reconstruction	2 (5.3)	3 (2.1)	
Not done breast Cancer surgery	17 (29.8)	47 (32.9)	
More than one	8 (14)	12 (8.4)	
**Other types of cancer**			0.100 *
Yes	3 (5.3)	7 (4.9)	
No	54 (94.7)	136 (95.1)	
**Did you have practices**			0.283
Yes	37 (64.9)	81 (56.6)	
No	20 (35.1)	62 (43.4)	
**Type of Practices**			0.037 *
Physical exercise and activity	2 (3.5)	19 (13.3)	
Social support	6 (10.5)	15 (10.5)	
Self-care and mindfulness	19 (33.3)	51 (35.7)	
More than one	23 (40.4)	54 (37.8)	
Other	7 (12.3)	4 (2.8)	

* Fisher exact test.

**Table 6:** Multiple Linear Regression Analysis of Mean Stress Scores by Demographic and Clinical Characteristics of Participants (*n* = 200).

Model	Unstandardized Coefficients		Standardized Coefficient β	t	*p*-Value	95.0% CI for B	
	B	Std. Error				Lower Bound	Upper Bound
(Constant)	29.801	5.903		5.049	<0.001	18.148	41.454
**Age**	−0.179	0.071	−0.225	−2.501	0.013	−0.319	−0.038
**Education** (Ref no education)							
Primary	4.067	3.307	0.184	1.230	0.220	−2.461	10.595
Secondary	2.099	3.395	0.080	0.618	0.537	−4.602	8.801
High school	3.652	3.092	0.224	1.181	0.239	−2.453	9.756
Diploma	4.119	3.442	0.179	1.197	0.233	−2.676	10.915
Bachelor	3.263	3.268	0.211	0.998	0.320	−3.189	9.714
Master	−0.204	5.626	−0.003	−0.036	0.971	−11.312	10.903
**Marital status** (Ref: Divorced)							
Single	0.455	2.781	0.022	0.164	0.870	−5.035	5.945
Married	−1.834	2.133	−0.112	−0.860	0.391	−6.045	2.376
Widow	1.732	3.234	0.054	0.536	0.593	−4.652	8.117
**Having children** (Ref: Yes)	2.873	1.893	0.165	1.518	0.131	−0.864	6.610
**Working hours per week** (Ref: Not working)							
Full duty (more than 20 h)	0.278	1.625	0.015	0.171	0.864	−2.929	3.486
Part time (less than 20 s/week)	0.471	2.394	0.016	0.197	0.844	−4.256	5.197
**Time diagnosis/month**	0.015	0.016	0.083	0.965	0.336	−0.016	.046
**Stages of cancer** (**Ref** Stage I)							
Stage-II	2.004	1.712	0.112	1.171	0.243	−1.376	5.384
Stage-III	0.508	1.766	0.027	0.288	0.774	−2.978	3.994
Stage-IV	4.649	2.391	0.167	1.944	0.054	−0.071	9.370
I don’t know	1.246	1.632	0.079	0.764	0.446	−1.975	4.467
**Type of Therapy Taken** (**Ref:** Chemotherapy)							
Radiotherapy	−0.879	2.430	−0.030	−0.362	0.718	−5.675	3.918
Hormonal	1.419	2.002	0.061	0.709	0.479	−2.533	5.370
More than one therapy	−0.209	1.353	−0.014	−0.154	0.877	−2.880	2.462
**Type of Surgery** (**Ref:** No breast surgery)							
Complete mastectomy	−0.136	1.546	−0.009	−0.088	0.930	−3.188	2.917
Partial mastectomy	1.777	1.847	0.087	0.962	0.337	−1.870	5.424
Nipple sparing surgery	−0.767	2.531	−0.025	−0.303	0.762	−5.764	4.231
Breast reconstruction	2.996	3.614	0.064	0.829	0.408	−4.140	10.131
More than one	−2.456	2.326	−0.101	−1.056	0.293	−7.049	2.137
**Engagement in stress-management practices (Ref: Yes)**	−1.684	1.724	−0.113	−0.977	0.330	−5.088	1.720
**Type of Practice** (Ref: More than one practice)							
Physical exercise and activity	1.060	2.146	0.044	0.494	0.622	−3.176	5.296
social support	0.035	2.231	0.001	0.016	0.988	−4.369	4.439
self-care and mindfulness	0.695	1.770	0.045	0.392	0.695	−2.800	4.189
Other	−2.484	2.850	−0.077	−0.871	0.385	−8.110	3.143

B = unstandardized coefficient; β = standardized coefficient; CI = confidence interval.

## Data Availability

The datasets used and/or analyzed during the current study are available from the corresponding author on reasonable request.
